# Remote diffusion-weighted imaging lesions in spontaneous intracerebral hemorrhage: research progress and new perspectives

**DOI:** 10.3389/fneur.2026.1748323

**Published:** 2026-06-26

**Authors:** Lingjia Xu, Yang Zhou, Feng Zhou, Guoping Fu

**Affiliations:** Department of Neurology, Shaoxing Second Hospital, The Second Affiliated Hospital of Shaoxing University, Shaoxing, Zhejiang, China

**Keywords:** diffusion-weighted imaging (DWI), intracerebral hemorrhage (ICH), prognosis, small vessel disease, stroke

## Abstract

Spontaneous or primary intracerebral hemorrhage (ICH) is considered the most severe form of stroke. Previous studies have focused mostly on local and perilesional changes in hematomas while paying less attention to damage in distant neural tissue. With the widespread use of magnetic resonance imaging (MRI) technology, an increasing number of studies have revealed that diffusion-weighted imaging (DWI) hyperintense lesions with low apparent diffusion coefficient (ADC) hypointensity can appear in remote areas away from the hematoma after ICH, indicating the presence of ischemic injury. However, the mechanism underlying their development remains unclear, and there is currently a lack of systematic reviews on the factors influencing remote DWI (R-DWI) lesions and their relationship with ICH prognosis. Additionally, some research findings are contradictory. Therefore, this review aims to systematically summarize the factors influencing R-DWI lesions after ICH and explore their predictive indicators, prognostic associations, and potential mechanisms.

## 1 Introduction

Spontaneous intracerebral hemorrhage (ICH) is the second leading cause of stroke after cerebral infarction, with exceptionally high incidence, disability, and mortality rates (1). Current research on ICH has primarily focused on local lesions and perilesional tissues, as well as predicting hematoma expansion. In contrast, relatively few studies have examined injury to remote neural tissue (2). With the advancement of magnetic resonance imaging (MRI) technology and its increasing clinical application in patients with ICH, numerous studies have revealed that after ICH, hyperintense lesions on diffusion-weighted imaging (DWI) can be observed in remote areas of the brain (3–5). These lesions are generally regarded as ischemic injuries occurring remotely from the hematoma, which may worsen the condition of the patient, hinder rehabilitation, and contribute to poor outcomes in patients with ICH (6). Because remote DWI (R-DWI) lesions may associated with poor prognosis, reducing their occurrence through modification of risk factors might represent a potential strategy for improving ICH outcomes.

R-DWI lesions in ICH are defined as areas that exhibit hyperintensity on MRI DWI sequence and corresponding hypointensity on apparent diffusion coefficient (ADC) map, located at least 10 mm away from the hematoma margin (7). These lesions are typically small and punctate, often located in the cortical or subcortical regions (8). Their pathogenesis remains unclear, but they are generally considered radiological manifestations of secondary remote ischemic injury following ICH. This review provides a comprehensive overview of the factors associated with R-DWI lesions after ICH, and discusses their predictive significance, correlation with clinical outcomes, and underlying mechanisms. To ensure a broad literature coverage, two reviewers (L. J. X. and Y. Z.) independently searched PubMed, Embase, and the Cochrane Library for relevant studies published in English using the following search terms: “cerebral hemorrhage” OR “intracerebral hemorrhage,” AND “diffusion-weighted imaging” OR “diffusion-weighted magnetic resonance imaging”. The study selection process is illustrated in Supplementary Material 1.

## 2 Heterogeneous mechanisms of R-DWI lesions after ICH

**Figure 1:**
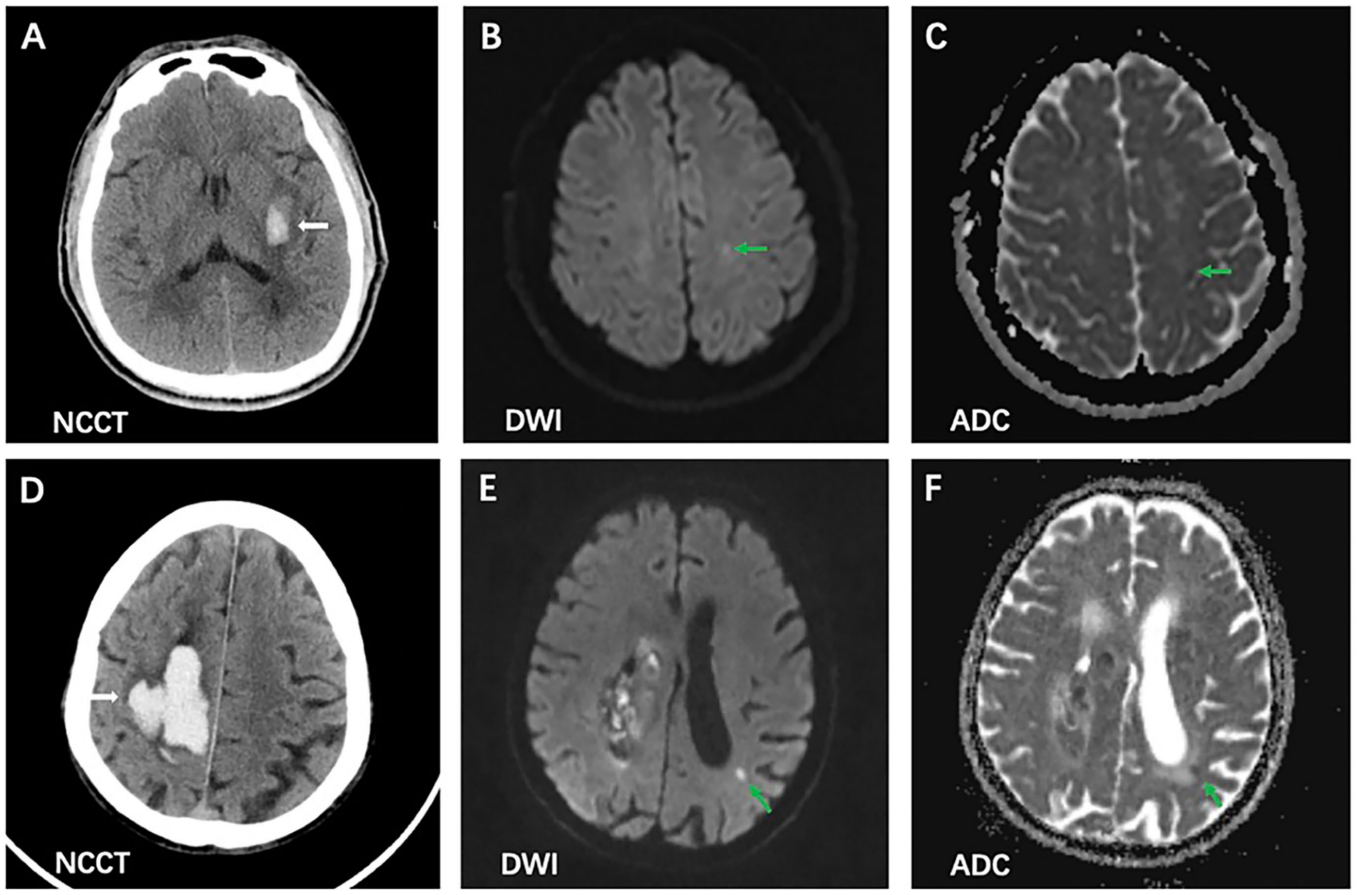
Remote DWI lesions in ICH. **(A–C)** Hypertensive ICH. Left basal ganglia hemorrhage, small punctate DWI hyperintensity in left parietal lobe with corresponding hypointensity in apparent diffusion coefficient (ADC) map. **(D–F)** Probable cerebral amyloid angiopathy-related ICH. Right parietal lobe hemorrhage, small punctate DWI hyperintensity next to the posterior horn of the left lateral ventricle with corresponding hypointensity in ADC map. NCCT, non-contrast computed tomography; DWI, diffusion-weighted imaging; ADC, apparent diffusion coefficient; ICH, intracerebral hemorrhage; white arrows indicate ICH lesions, green arrows indicate remote DWI lesions.

The frequency of R-DWI lesions on MRI in patients with spontaneous or primary ICH varies across studies and is approximately 11–50% (Table 1) (9, 10). Patients with R-DWI lesions are typically classified into three groups: those with hypertensive arteriopathy and cerebral amyloid angiopathy (CAA), and those with coexisting hypertensive arteriopathy and CAA (11). Representative images were shown in Figure 1.

**Table 1:** Characteristics of studies on R-DWIL in ICH patients.

Study (year)	Sample size	Study design	Male (*%*)	Age (year)	DM (*%*)	HTN (*%*)	MRI timing	ICH etiology (*%*)			R-DWIL (+) (*%*)	Presume etiology in R-DWIL(+) patients (*%*)		
							(d/h)	CAA	HTN	other		CAA-ICH	HTN-ICH	Other cause
Arsava et al. (2013) (49)	86	Prospective	59.3	64	19.8	77.9	≤14d	30.2			17.4	33.4		
Auriel et al. (2012) (23)	392	Retrospective	51	72.8	17.9	71.7	≤7 d	15.2	17.2		18.2	58.9	28.0	
Buletko et al. (2018) (101)	119	Retrospective	58	72	27.7	79.8	-				23.7			
Butcher et al. (2025) (48)	162	Clinical trail	53.7	71	22.8	72.2	mean 51.6 h				48.8			
Chen et al. (2020) (93)	160	Retrospective	62.5	57.1	9.4	68.1	≤28d				16.9			
Garg et al. (2012) (41)	95	Prospective	49	64.1	24.2	78.9	0.8–7.5d	15	62		41			
Garg et al. (2020) (6)	121	Prospective	59.5	56.5	19	84.3	≤3d	6.6	93.4		49.6	6.7	93.3	
Gioia et al. (2015) (73)	117	Retrospective	52.1	65.6		65	1–5 d	9.4	45.7		14.5			
Goeldlin et al. (2025) (12)	644	Multicenter cohort	54.1	73	15.3	75.1	mean 2d	32.3			25	32.5		
Gregoire et al. (2011) (102)	114	Multicenter case–control	88		16.7	77.2	55% < 7 d	34			13	60.7		
Hosoki et al. (2025) (14)	872	Retrospective	55.8	70.9	16.7	89.6	-	10.9	74.8		13.1	8.8	79.8	
Hou et al. (2024) (85)	408	Retrospective	73.3	56.8	16.7	58.8	≤14d				21.8			
Kimberly et al. (2009) (103)	78	Retrospective case–control	41	78.2	6	58	48.7% <7d	100	0	0	15			
Kang et al. (2012) (4)	97	Prospective	71	59.1	14.4	84.5	≤3d	0	100	0	26.8	0	100	0
Kidwell et al. (2017) (2)	600	Prospective	55.5	60.8		79.2	mean 1.6d				26.5			
Li et al. (2020) (66)	163	Retrospective	60.7	62.3	11	74.8	5-8d				19.0			
Lusk et al. (2023) (104)	1,167	Retrospective	59	57	26	80	-				26.7			
Menon et al. (2012) (42)	138	Prospective	54.6	59.8		84	mean 2d	1.5	62		35			
Murthy et al. (2020) (9)	1752	Pooled Analysis	58.2	60.8	26.1	81.6	-				31.3			
Poyraz et al. (2025) (105)	190	Prospective	50	66.6			mean 2/4d				32			
Prabhakaran et al. (2010) (3)	118	Retrospective	47	59.6	26.3	77.1	1-3d	11.9	70.3	13.6	23		84.7	
Revel et al. (2019) (31)	246	Retrospective	59	71.4		66.7	≤10d				15.4			
Rivera-Lara et al. (2024) (74)	300	Subgroup Analysis	59.3		31.3	96.7	≤7d				59			
Ridha et al. (2024) (106)	877	Prospective	57.5	60.5	24.8	81.5	mean 3.19d				25.9			
Roh et al. (2023) (96)	917	Prospective	58	60	25	78	mean 22 h				27			
Shoamanesh et al. (2022) (47)	171	Subgroup Analysis	62.6	62	22.5	78	≤10d				25			
Tsai et al. (2014) (11)	153	Prospective	60.1	63.8	20.9	90.8	≤14d				11.1			
Wiegertjes et al. (2021) (100)	247	Randomized trial	68.8	75.7			mean 57d	17.4			30	28.3		
Wang et al. (2025) (86)	245	Retrospective	64.5	62	9.4	63.3	mean 72 h				18.78			
Wu et al. (2015) (13)	201	Retrospective	60	70.5	22.9	71.6	≤30d	34.8	55.2	10	27.9	41.1	50.0	8.9
Xu et al. (2018) (107)	126	Prospective	56.3	61.8	9.5	72.2	mean 6d				23			
Xu et al. (2019) (15)	344	Prospective	61.3	60.3	12.2	68.9	mean 6d	18.3	58.7	23.0	16.6	19.3	63.2	17.5
Xu et al. (2022) (56)	375	Retrospective	65.3	61.0	14.3	75.7	mean 5d				17.3			
Ye et al. (2018) (7)	222	Prospective	59.9	59.9	8.1	73	≤28d				18.5			
Ye et al. (2020) (82)	288	Prospective	61.8	59.7	8.7	68.8	≤14d	17	59.4	23.6	16	17.4	60.9	21.7
Ye et al. (2021) (83)	345	Prospective	64.6	61.1	18.8	76.8	≤14d	11.6	70.4	18.0	15.7	7.4	79.6	13.0
Zhu et al. (2025) (72)	229	Retrospective	65.9	63	15.7	79.9	mean 6d				19.7			

R-DWIL, remote diffusion-weighted imaging lesions; d, days; h, hours; DM, diabetes mellitus; HTN, hypertension; ICH, intracerebral hemorrhage; CAA, cerebral amyloid angiopathy.

The pathogenesis of R-DWI lesions after ICH is likely heterogeneous across patients, which may explain the conflicting findings in the literature. Based on current evidence, three main mechanisms have been proposed: simultaneous vessel rupture and occlusion due to the severity of underlying cerebral small vessel disease (CSVD); overly aggressive blood pressure (BP) lowering, although this may be confounded by the fact that patients with higher admission BP may have more severe CSVD; prothrombotic and/or inflammatory processes after ICH that predispose to subsequent ischemic events. The following sections review the evidence for each mechanism, and summary tables (Tables 2–4) consolidate key study results.

**Table 2:** Key studies on the association between CSVD and R-DWIL.

Study (year)	RDWIL definition	Main findings
Goeldlin et al. (2025) (12)	SVD markers were assessed according to STRIVE criteria, R-DWIL were defined <20 mm in diameter with a minimum distance of 5 mm from the hematoma	18.4% already had R-DWIL on admission MRI, higher SVD and CAA burdens were associated with presence of R-DWIL
Hosoki et al. (2025) (14)	R-DWIL excluded within or a 10 mm rim around the hematoma	Associated with CSVD markers and higher CSVD severity score
Revel et al. (2019) (31)	R-DWIL in close proximity (<10 mm) to the hematoma were excluded, CSS was visually assessed	CSS severity and WMHs degree were independent predictors of R-DWIL
Wu et al. (2015) (13)	R-DWIL were defined <10 mm in diameter with a distance over 20 mm from the hematoma	High EPVS and larger hematoma volume were independent predictors
Xu et al. (2019) (15)	R-DWIL were defined <20 mm in diameter with a distance over 20 mm from the hematoma	Associated with higher CSVD burden and mixed CMBs

CSVD, cerebral small vessel disease; R-DWIL, remote diffusion-weighted imaging lesions; SVD, small vessel disease; CAA, cerebral amyloid angiopathy; CSS, cortical superficial siderosis; WMHs, white matter hyperintensities; EPVS, enlarged perivascular spaces; CMBs, cerebral microbleeds.

**Table 3:** Key studies on BP parameters and R-DWIL.

Study (year)	BP assessment	Main findings
Arsava et al. (2013) (49)	Admission BP	Elevated admission mean-arterial pressure significantly associated with R-DWIL
Butcher et al. (2025) (48)	Early intensive (target 140 mmHg) vs. standard BP lowering	Early intensive treatment did not increase number or volume of R-DWIL
Garg et al. (2012) (41)	BP reduction	R-DWIL associated with greater BP decrease and worse 3-month outcomes
Gioia et al. (2015) (73)	Serial BP measurements	Perihematomal edema volume, not BP reduction, predicted ischemic threshold
Shoamanesh et al. (2022) (47)	Intensive vs. standard BP lowering	No significantly higher frequency of R-DWIL detected
Xu et al. (2022) (56)	BP variability	Higher BP variability associated with 3-8-fold increased risk of R-DWIL

R-DWIL, remote diffusion-weighted imaging lesions; BP, blood pressure.

**Table 4:** Key studies on prothrombotic/inflammatory markers and R-DWIL.

Study (year)	Markers	Main findings
Li et al. (2020) (66)	NLR	Elevated NLR independently associated with R-DWIL
Rocha et al. (2021) (39)	CME by TCD	Higher CME incidence in ICH
Zhu et al. (2025) (72)	FAR	Elevated FAR1 and FAR2 associated with R-DWIL; FAR2 with poor 1-month outcome

R-DWIL, remote diffusion-weighted imaging lesions; CME, cerebral microembolism; TCD, transcranial doppler; ICH, intracerebral hemorrhage; NLR, neutrophil-to-lymphocyte ratio; FAR, fibrinogen-to-albumin ratio.

### 2.1 Underlying CSVD

A growing body of evidence indicates that R-DWI lesions are closely linked to the severity of pre-existing CSVD. Importantly, a recent prospective study observed that 18.4% of patients already had R-DWI lesions on MRI at the time of ICH admission (12), suggesting that some of these lesions are not caused by the acute ICH itself but rather reflect the underlying microvascular pathology. Patients with R-DWI lesions consistently show a higher burden of CSVD markers, including cerebral microbleeds (CMBs), white matter hyperintensities (WMHs), lacunes, and enlarged perivascular spaces (EPVS) (13–15). Mixed CMBs (both lobar and deep) have been significantly associated with R-DWI lesions, whereas the association with strictly lobar or deep CMBs is less consistent (15). High-grade EPVS, particularly in the lateral paraventricular region, and large hematoma volume are independent predictors of R-DWI lesions (13). Moreover, the total burden of WMHs, including both deep and periventricular WMHs, may reliably predict the presence of R-DWI lesions (16).

The pathophysiological link between CSVD and R-DWI lesions is likely multifactorial. In CAA, progressive accumulation of *β*-amyloid in the walls of leptomeningeal and cortical arterioles leads to vessel wall rupture, impaired vascular remodeling, and luminal occlusion, which can cause cerebral microinfarcts (CMIs) (17–19). Histopathological studies have shown that CMIs and CMBs share common features, including vascular wall rupture, impaired vascular remodeling processes, vascular wall thickening with increasing CAA severity, and blood–brain barrier (BBB) dysfunction (20, 21). In hypertensive arteriopathy, advanced vascular changes similarly predispose to both hemorrhage and microinfarction (18, 19). Notably, some studies found no significant difference in R-DWI lesions incidence between CAA-related ICH and hypertension-related ICH (10, 22), suggesting that the underlying CSVD burden, rather than its specific etiology, may be the key driver.

The distribution of R-DWI lesions does not strictly follow the expected topography of the underlying vasculopathy. In lobar ICH (predominantly CAA), CAA-related hemorrhage tends to be most severe in the superficial cortex, especially posterior cortical areas, rather than in the frontal lobes (23–25). The superficial cortical distribution of R-DWI lesions also suggests, to some extent, involvement of vascular border-zone or “watershed” regions. Unexpectedly, posterior cortical R-DWI lesions were predominant in patients with deep ICH, who may have hypertension-related small vessel disease (SVD), which primarily affects the basal ganglia, thalamus, and deep brainstem perforating arteries (26). The apparent discrepancy between SVD pathology and the location of SVD-associated R-DWI lesions suggests that the pathophysiologic processes leading to these microinfarctions have not yet been fully identified in these cases.

The CSVD is a broad term for a group of microvascular brain lesions (27), substantial evidence indicates that R-DWI lesions are associated with CSVD (14). However, most of these studies only focused on one or two CSVD imaging markers, and few evaluated all markers or the total CSVD burden in a single study. Moreover, fewer studies investigated the relationship between the distribution location of CSVD and R-DWI lesions. A study of baseline MRI data from 344 patients with ICH demonstrated that patients with R-DWI lesions had a higher frequency of CSVD imaging markers (CMBs, WMHs, and lacunes) and a greater overall burden of CSVD (15). This study also found that mixed CMBs, rather than strictly lobar or deep CMBs, were significantly associated with the presence of R-DWI lesions (15). However, Wu et al. previously observed that the prevalence of R-DWI lesions was higher in patients with lobar CMBs than in those with deep or mixed CMBs, significant associations were also found between lobar and deep mixed lacunes and R-DWI lesions (13). High EPVS, particularly lateral paraventricular EPVS and large hematoma volume, are independent predictors of R-DWI lesions after ICH (13). This may also suggest the role of hypertension-related SVD in the etiology of R-DWI lesions. Moreover, high-grade WMHs, as well as total WMHs, deep WMHs and periventricular WMHs may be reliable predictors for the indication of R-DWI lesions. The association between R-DWI lesions and WMHs load suggests that at least some white matter changes may be due to cumulative microinfarcts or chronic hypoperfusion (16). Another potential mechanism of damage to white matter by cortical and leptomeningeal vascular vessels affected by CAA is retention of β-amyloid in the perivascular space, which impairs the interstitial drainage pathway of white matter (28, 29). In summary, CSVD distribution correlates with R-DWI lesions, CAA typically causes lobar CSVD, whereas hypertension causes deep or mixed CSVD. Further studies are needed to confirm this association. Besides, some genetic CSVD diseases, such as Cerebral Autosomal Dominant Arteriopathy with Subcortical Infarcts and Leukoencephalopathy, may be more likely to develop R-DWI lesions (30).

Several new CSVD-related studies have demonstrated that R-DWI lesions occur more frequently in patients with acute lobar ICH than in those with deep ICH. The severity of cortical superficial siderosis (CSS) and the degree of WMH in CSS are independent predictors of the existence of R-DWI lesions, suggesting that CSS may be involved in the pathogenesis of R-DWI lesions associated with acute ICH (31, 32).

The distinct clinical manifestations of R-DWI lesions can also be considered CMIs, which are common findings in patients with ICH (33). CMIs are also frequent in other types of stroke and vascular diseases, ranging from 15% in patients with transient ischemic attack or ischemic stroke to 30% in those with heart disease (34, 35). CMIs are more frequent in patients with CAA-related ICH, with rates of up to 60% (36, 37). Although the presence of CMIs is not specific to ICH, the mechanism of CMIs may be related to the occurrence of R-DWI lesions following ICH. The etiology of CMIs is unclear but appears to be related to advanced age and cardiovascular disease, and is also considered a possible manifestation of CSVD; however, large vessel atherosclerosis also plays a role (38). The mechanism of these small infarctions is thought to involve hypoperfusion, BP variability, and microemboli (39, 40). Table 2 summarizes key studies on the association between CSVD burden and R-DWI lesions.

### 2.2 BP reduction and cerebral autoregulation

Acute BP lowering is standard management for ICH, but overly aggressive reduction may precipitate cerebral ischemia, especially in patients with impaired autoregulation. Previous studies have demonstrated that R-DWI lesions are associated with a significant decrease in BP within 24 h, although the mechanism underlying the greater BP decreases in patients with ICH with R-DWI lesions is unclear (3, 41, 42). Garg et al. (41) found that R-DWI lesions were associated with greater BP decreases and worse functional outcomes. When BP is acutely lowered, intracranial pressure rises and may trigger a prethrombotic and proinflammatory cascade, increasing the frequency of ischemic episodes (11). A decrease in BP below the lower limit of autoregulation after acute ICH is also associated with cerebral hypoperfusion and ischemia (43). Significant reduction in BP during the acute phase of ICH may be related to the disruption of cerebral autoregulation, which is the ability to regulate cerebral blood flow resistance to minimize deviations in cerebral blood flow and maintain normal brain function (44, 45). The formation of hematoma and brain edema may lead to increased intracranial pressure, causing an imbalance in cerebral blood flow autoregulation and resulting in cerebral hypoperfusion, thereby leading to cerebral ischemic lesions (46). Additionally, due to the dysfunction of small vessels, even if overall cerebral blood flow can be maintained, local changes in cerebral blood flow and insufficient perfusion may still occur, triggering cerebral ischemia, especially in areas with dysfunction of distal cerebral small vessels (4).

However, other studies have demonstrated that R-DWI lesions are not associated with antihypertensive therapy but rather with elevated BP at admission (47, 48). Patients with R-DWI lesions had higher systolic, diastolic, and mean arterial BP levels at admission, as well as a greater burden of periventricular leukoaraiosis (49). Arsava et al. (49) found both mean arterial BP on admission and the Glasgow Coma Scale score remained significantly associated with the presence of R-DWI lesions. The etiology of R-DWI lesions involves underlying cerebral microvascular dysfunction. The effect of acute BP spikes on cerebrovascular vessels has long been recognized (50), sudden increases in BP can trigger vasospasm in the proximal cerebral arteries, contributing to the pathogenesis of reversible posterior leukoencephalopathy and reversible vasoconstriction syndrome (51, 52). Acute hypertension may also exert toxic effects on distal leptomeningeal arteries, leading to occlusive spasm and vessel necrosis (53). Consequently, elevated initial BP on admission is itself a potential precipitating factor for both ICH events and concomitant R-DWI lesions. The latest research on intensified antihypertensive treatment (target 140 mmHg) confirmed that early intensive BP lowering does not increase the number or volume of ischemic foci (48).

Blood pressure variability (BPV) has been independently associated with R-DWI lesions within 24 h of ICH onset (54). Several BPV indices, including admission systolic blood pressure (SBP), maximum SBP, minimum SBP, and SBP range (maximum-minimum), more accurately represent physiological fluctuations (55). Xu et al. (56) reported that BPV values in the highest quintile were associated with a 3–8-fold increased risk of R-DWI lesions compared with the lowest quintile. This finding suggests that efforts should be made to stabilize BP during the acute phase rather than strictly maintaining low BP (43).

The INTERACT3 Trial showed that intensive BP reduction (target 130–140 mmHg) within 6 h of ICH onset improves outcomes (57). In summary, BPV within 24 h after the onset of ICH was independently associated with R-DWI lesions (56). This finding implies that efforts should be made to stabilize BP during the acute phase of ICH rather than strictly maintaining low BP (43). However, further research on longer-term and more comprehensive BP monitoring after the acute phase of ICH is needed to confirm the effects of BP not only on hematoma growth and neurological outcomes after ICH but also on remote neural tissues. Individualized, autoregulating informed antihypertensive targets may provide a new paradigm for the acute management of ICH, which requires further investigation. Table 3 summarizes key studies on the relationship between BP parameters and R-DWI lesions.

### 2.3 Prothrombotic and inflammatory processes after ICH

The exact pathophysiological mechanism of R-DWI lesions remains uncertain and may be the result of multiple mechanisms. Firstly, the hypercoagulable state caused by acute ICH, as well as the fragile microvascular state and underlying CSVD in patients with ICH, can easily lead to increased intracranial pressure and autoregulatory dysfunction, making the brain vulnerable to BP fluctuations and resulting in low cerebral blood flow perfusion (22, 39). Another possible cause underlying R-DWI lesions is cerebral microembolism (CME). The incidence of CME was higher among patients with ICH than among the controls (39). The source of CME remains uncertain but appears to be associated with vascular risk factors, atherosclerosis, and cardiac hypertrophy (39). Further studies are needed to investigate the embolic origin of CME in ICH and its clinical and radiologic significance.

The inflammatory response after acute ICH occurs rapidly and involves complex interactions between cells of the resident brain tissue and the peripheral immune system (58, 59). Experimental and clinical evidence suggest that neuroinflammation plays a key role in secondary brain injury following ICH and may lead to adverse outcomes (60). The role of neutrophils in promoting blood coagulation and thrombosis may increase the risk of developing ischemic lesions (61). The neutrophil-to-lymphocyte ratio (NLR) is a more reliable marker of the inflammatory state of the individual than the total white blood cell and neutrophil counts (62). Cerebral microvascular injury is already present during ICH, and an elevated NLR promotes the externalization of neutrophils to decondensed nucleosomes and granule proteins, forming neutrophil extracellular traps, which can lead to microvascular thrombosis (63). Overall, a higher NLR indicates a greater tendency toward coagulation or microemboli formation. In patients with ICH, an elevated NLR is strongly associated with mortality risk and is also associated with stroke severity and perihematomal edema growth (64). Recent significant observations indicate that the NLR is an independent predictor of delayed cerebral ischemia in patients with subarachnoid hemorrhage (SAH) (65). Li et al. revealed that the NLR is independently associated with the occurrence of R-DWI lesions in spontaneous ICH (66).

Another inflammatory marker is the fibrinogen-to-albumin ratio (FAR). Fibrinogen (FIB) is a liver-synthesized plasma coagulation factor that regulates blood viscosity and platelet activation and contains critical binding sites for thrombin and platelet glycoproteins IIb/IIIa (67). Thrombin cleaves FIB into fibrin monomers, and FIB cross-links activated platelets via glycoprotein IIb/IIIa to drive aggregation (68). It also serves as a marker of systemic inflammation, and fibrin–neuron interactions increase Interleukin-6 levels, oxidative stress, and BBB permeability (69, 70). Albumin (ALB), synthesized by the liver, exerts neuroprotective effects by reducing BBB permeability, inhibiting apoptosis, oxidation, and inflammation, and suppressing platelet aggregation and FIB activity (71). For this reason, FAR reflects thrombosis, inflammation, and disease progression more accurately than either marker alone, serving as a convenient biomarker for the coagulation-inflammation balance. Xu et al. recorded FIB, ALB, and FAR values on the first day of admission (designated FIB1, ALB1, and FAR1) and during the follow-up period, 3–7 days after admission (designated FIB2, ALB2, and FAR2) (72). They reported that elevated FAR1 and FAR2 were both associated with post-ICH R-DWI lesions and served as risk factors, and elevated FAR2 was associated with poor prognosis 1 month after ICH (72). Table 4 summarizes key studies on prothrombotic/inflammatory mechanisms and R-DWI lesions.

## 3 Other R-DWI lesions-related influencing factors

### 3.1 Index hematoma

The volume of hematoma and perihematomal edema may affect not only the primary hemorrhagic site of the ICH but also remote brain areas through secondary neural pathways (8). Studies have demonstrated that the larger the hematoma volume is, the greater the likelihood of R-DWI lesions (13, 42). Baseline hematoma volume, hematoma expansion, and perihematomal edema are associated with poor adverse outcomes in patients with ICH (73). Small R-DWI lesions within and beyond the perihematomal area are common in patients with ICH (14), and perihematomal R-DWI lesions are independently associated with larger hematoma volumes (73). The total volume of R-DWI lesions is also correlated with the size of the hematoma (74). According to the multivariable linear regression model adjusted for age, the baseline National Institutes of Health Stroke Scale score and perihematomal edema volume were the only independent predictors of the ischemic threshold after ICH (73). Among patients with R-DWI lesions, those with such lesions tended to have larger hematoma volumes than those without them, and they also exhibited greater median leukoaraiosis volumes (73).

### 3.2 Glycemic correlation

In rodent models, persistent hyperglycemia is associated with accelerated atherosclerosis through mechanisms involving systemic inflammation and plaque formation (75). In the context of stress-induced hyperglycemia, catecholamines and glucocorticoid levels increase, which can lead to microvasospasm, manifested by *in situ* small vessel occlusion (76). Studies in rat SAH models have also reported that hyperglycemia mediates cerebral vasospasm through the Nitric Oxide pathway (77). Clinically, poor glycemic control is associated with SVD. In addition to endothelial dysfunction, increased blood glucose levels and insulin resistance can lead to BBB dysfunction, facilitating the development of cerebral ischemia (78, 79). Hyperglycemia also fosters a prethrombotic environment through impaired fibrinolysis and platelet dysfunction, which can lead to acute thrombosis (80). The combination of progressive atherosclerosis in small cerebral vessels with a prethrombotic state provides the basis for acute R-DWI lesions, which may be further exacerbated by inflammation and thrombosis following ICH (42, 81) (these evidences also support the aforementioned third mechanism related to the R-DWI lesions).

In a prospective observational study, elevated admission blood glucose was identified as an independent risk factor for R-DWI lesions following ICH (6). Similarly, other studies have demonstrated that fasting blood glucose, stress-induced hyperglycemia and insulin resistance are associated with R-DWI lesions (7, 82, 83). However, strict glycemic control can result in systemic and cerebral hypoglycemia, leading to ischemia and secondary neuronal injury. These studies may have focused on a single blood glucose measurement at admission or at a fixed time point without dynamic glucose monitoring. Garg et al. (84) reported that, compared with patients in the R-DWI lesions-negative group, the patients in the R-DWI lesions-positive group received insulin infusions for a greater percentage of Intensive Care Unit admission time. Throughout hospitalization, the median blood glucose levels were significantly higher in patients with ICH with R-DWI lesions than in patients without, additionally, the presence of R-DWI lesions in patients with ICH was associated with higher mean longitudinal blood glucose throughout the hospital stay. This indicates that continuous hyperglycemia rather than dichotomous hyperglycemia is the risk factor for R-DWI lesions. In the study by Hou et al. (85), the average fasting blood glucose level of patients with R-DWI lesions was 7.23 mmol/L, among patients with R-DWI lesions, 39.3% had diabetes, further confirming a strong correlation between hyperglycemia and R-DWI lesions. Another indicator, triglyceride-glucose index was found no definitive correlation on R-DWI lesions in Wang’s study, however, they found the elevated high fasting glucose levels and hematoma site were significant predictors for R-DWI lesions after ICH (86).

### 3.3 Other serum indicators

**Figure 2:**
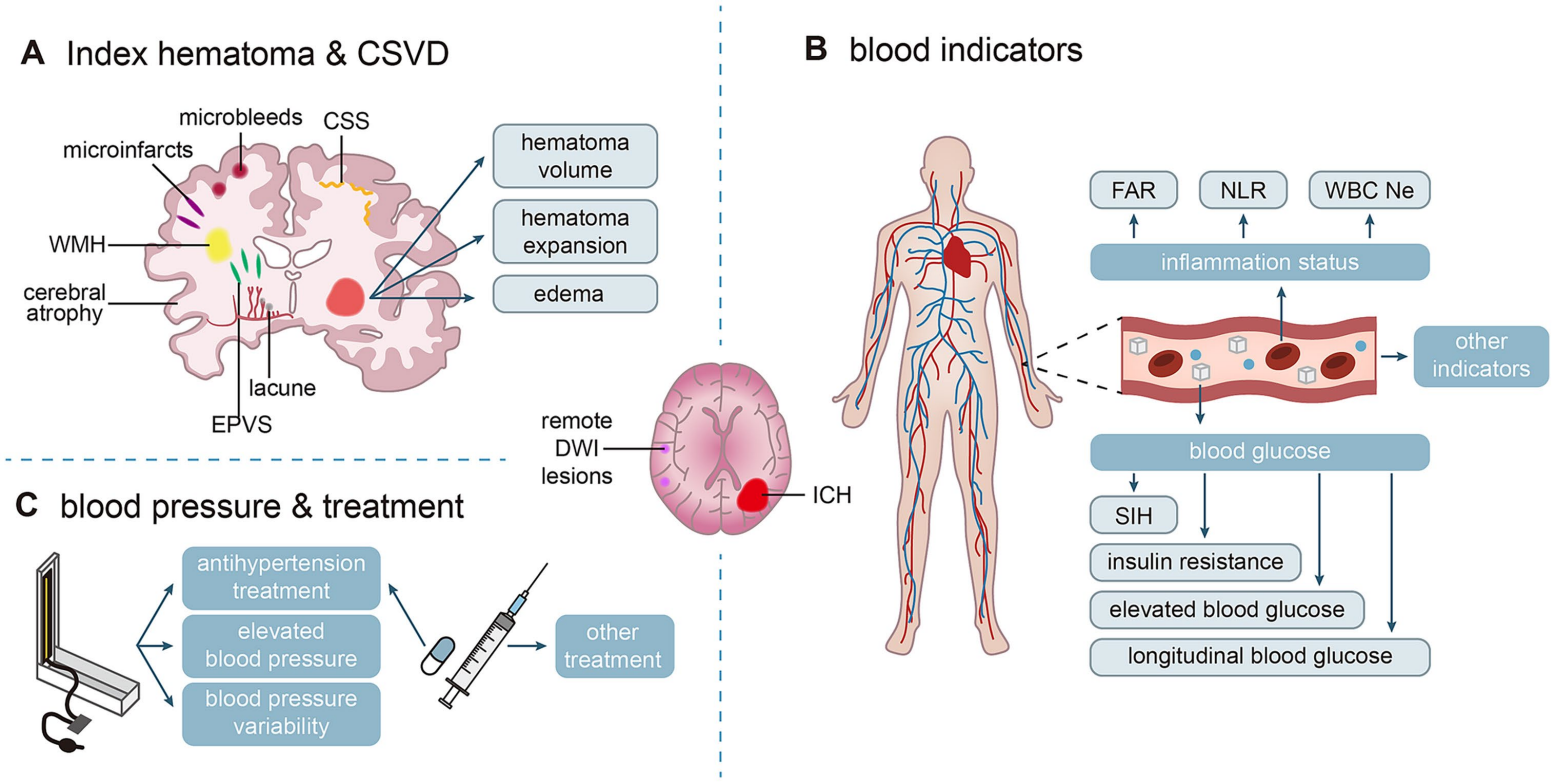
Remote DWI lesions-related influencing factors. **(A)** Index hematoma and CSVD. **(B)** Blood indicators. **(C)** Blood pressure and treatment. CSVD, cerebral small vessel disease; WMH, white matter hyperintensity; EPVS, enlarged perivascular space; CSS, cortical superficial siderosis; FAR, fibrinogen-to-albumin ratio; NLR, neutrophil-to-lymphocyte ratio; WBC, white blood cells; Ne, neutrophils; SIH, stress-induced hyperglycemia.

In addition to blood glucose, several potential serum biomarkers for R-DWI lesions have been identified. For example, studies have confirmed that lactate dehydrogenase (LDH) is related to cerebrovascular disease, and Li′s study revealed that LDH activity in brain tissue increases following ICH (87). Zhou’s study revealed lactate accumulation in the brain after ICH (88) and elevated serum LDH levels was detected in other studies with central nervous system diseases such as cerebral infarction and hypoxic–ischemic encephalopathy (89–91). Collectively, these studies confirmed the potential association between LDH and R-DWI lesions formation. Chu’s study revealed that serum LDH levels independently predict hematoma expansion and adverse outcomes (92). Similarly, a study by Chen et al. revealed a significant association between serum LDH levels and R-DWI lesions after spontaneous ICH (93). Higher serum LDH levels within 24 h following ICH onset may serve as a key predictor of the occurrence of R-DWI lesions. Furthermore, detecting LDH concentrations in cerebrospinal fluid within the first hour after stroke onset may have diagnostic and prognostic value, and the presence of LDH in cerebrospinal fluid may provide complementary information, such as the identification of more ischemia-related markers (94). R-DWI lesions-related influencing factors are shown in Figure 2.

## 4 R-DWI lesions and prognosis: contradictory outcomes

Whether the presence of R-DWI lesions predicts poor functional outcomes after ICH remains controversial, and current evidence does not uniformly support a harmful effect (95). Several studies have reported associations between R-DWI lesions and adverse outcomes. For instance, multivariate analysis by Garg et al. (41) showed that R-DWI lesions were associated with greater BP reduction, as well as increased disability and death at 3 months (6). Other studies have linked R-DWI lesions to worse 3-month modified Rankin Scale (mRS) scores and impaired post-discharge recovery (96). A recent meta-analysis including 3,575 patients from 10 studies reported an incidence of R-DWI lesions ranging from 11.1 to 49.6% (97). In six of these studies, R-DWI lesions were associated with an increased risk of poor functional outcome (mRS 3–6), and some also suggested a link with ICH recurrence; however, the relationships between R-DWI lesions and mortality, ischemic stroke, or hematoma volume remained inconsistent (97). Conversely, other well-designed studies have found no independent association. Kang et al. reported that R-DWI lesions were not independently associated with 3-month functional outcomes (4). Similarly, a multicenter Swiss stroke study revealed that R-DWI lesions were not significantly associated with 3-month functional prognosis, recurrent ICH, or ischemic stroke (12). These contradictory findings highlight that the prognostic significance of R-DWI lesions is likely context-dependent, and that they should not be treated as uniformly harmful.

One potential explanation for the inconsistent associations between R-DWI lesions and outcomes is that ICH prognosis is driven predominantly by the severity of the ICH itself (e.g., hematoma volume, location, intraventricular extension, perihematomal edema), such that any additional effect of R-DWI lesions on outcome may be relatively modest and harder to detect in multivariable models. The modest effect size may only become apparent in very large cohorts or specific subgroups.

A critical nuance in interpreting these lesions is their uncertain pathological fate. Emerging evidence suggests that R-DWI lesions may serve as surrogate markers of SVD activity rather than permanent infarction. In a follow-up study, Tsai et al. found that patients with good functional outcomes were more likely to have R-DWI lesions that were not visible on follow-up MRI (11). After 3 months, regression of R-DWI lesions was associated with good functional outcomes (98). In that study, more than half of R-DWI lesions in ICH patients did not progress to visible chronic infarction. Poor functional outcome was predicted only when R-DWI lesions eventually evolved into final infarction, illustrating that R-DWI lesions may represent ischemic changes in the setting of SVD and do not always indicate permanent tissue injury. This phenomenon can be partly explained by the pseudonormalization of ADC values during the subacute stage. As DWI hyperintensity transitions to isointensity, the lesion may become undetectable on standard DWI, leading to underestimation of the true ischemic burden. Thus, the absence of a visible chronic infarct on follow-up does not necessarily equate to absence of prior tissue injury. This evidence provides a new perspective on the clinical significance of R-DWI lesions. They may serve as a dynamic marker of SVD activity, and their resolution on follow-up imaging may reflect milder vascular insults or transient occlusion with early reperfusion (14, 99). Among ICH survivors, stroke recurrence has been linked to recurrent hemorrhage rather than ischemic stroke (100). Further research is needed to elucidate the natural course of R-DWI lesions and their underlying mechanisms as a microvascular marker associated with recurrent ICH. Table 5 summarizes key studies on the prognostic value of R-DWI lesions.

**Table 5:** Studies on the prognostic value of R-DWIL.

Study (year)	Outcome	Main findings
Garg et al. (2012) (41)	3-month mRS, death	R-DWIL associated with worse outcomes
Garg et al. (2020) (6)	3-month disability, death	R-DWIL associated with greater disability and death
Goeldlin et al. (2025) (12)	3-month functional outcome	No independent association
Kang et al. (2012) (4)	3-month functional outcome	No independent association
Li et al. (2024) (97)	Poor functional outcome (mRS 3–6)	Pooled association; inconsistent for mortality/ischemic stroke
Roh et al. (2023) (96)	Discharge disposition, 3-month mRS	R-DWIL associated with severe disability/death and independently with poor outcome
Tsai et al. (2014) (11)	Lesions regression on follow-up MRI	>50% lesions disappeared; regression associated with good functional outcome
Wiegertjes et al. (2021) (100)	Recurrent stroke	R-DWIL associated with recurrent ICH, not with ischemic stroke

R-DWIL, remote diffusion-weighted imaging lesions; mRS, modified ranking scale; ICH, intracerebral hemorrhage.

## 5 Limitations

Several limitations should be acknowledged in interpreting the findings of this review. First, there was substantial heterogeneity across the included studies regarding the timing of MRI acquisition (ranging from acute to subacute phases), which may influence the detection rate and characteristics of R-DWI lesions. Second, the definition of R-DWI lesions varied considerably among studies, including differences in minimum distance from the hematoma, lesion size thresholds, and methods for excluding chronic ischemic changes, leading to potential misclassification. Third, the etiological classification of ICH was not uniform across studies (e.g., deep versus lobar, hypertensive versus cerebral amyloid angiopathy), which may affect the association between R-DWI lesions and clinical outcomes. Fourth, the extent of adjustment for potential confounders (such as hypertension, anticoagulation use, and baseline hematoma volume) was inconsistent, and many studies were based on observational data with inherent residual confounding. Finally, most of the available evidence is derived from observational cohorts, and data from randomized controlled trials are lacking; therefore, causal inferences cannot be made. Future prospective studies with standardized MRI protocols, uniform definitions of R-DWI lesions, and rigorous adjustment for confounders are needed to validate the prognostic and therapeutic implications discussed in this review.

## 6 Conclusion

In summary, R-DWI lesions occurring remote from the hematoma are not uncommon and may represent potential markers of SVD activity. The mechanisms underlying these lesions after ICH remain unclear; however, factors such as the primary hematoma effect, inflammation status, CSVD, and elevated blood glucose are considered influential factors. Autoregulation-informed antihypertensive strategies may offer a new approach to the acute management of ICH. Although inconsistencies persist regarding the prognostic impact of R-DWI lesions, understanding their pathophysiological basis is crucial for acute treatment optimization and improving patient outcomes.
