# Supplementary figures and images for: Remote diffusion-weighted imaging lesions in spontaneous intracerebral hemorrhage: research progress and new perspectives
Source: Front Neurol. 2026 Jun 26;17:1748323. doi: 10.3389/fneur.2026.1748323 (PMC13349777; doi:10.3389/fneur.2026.1748323)

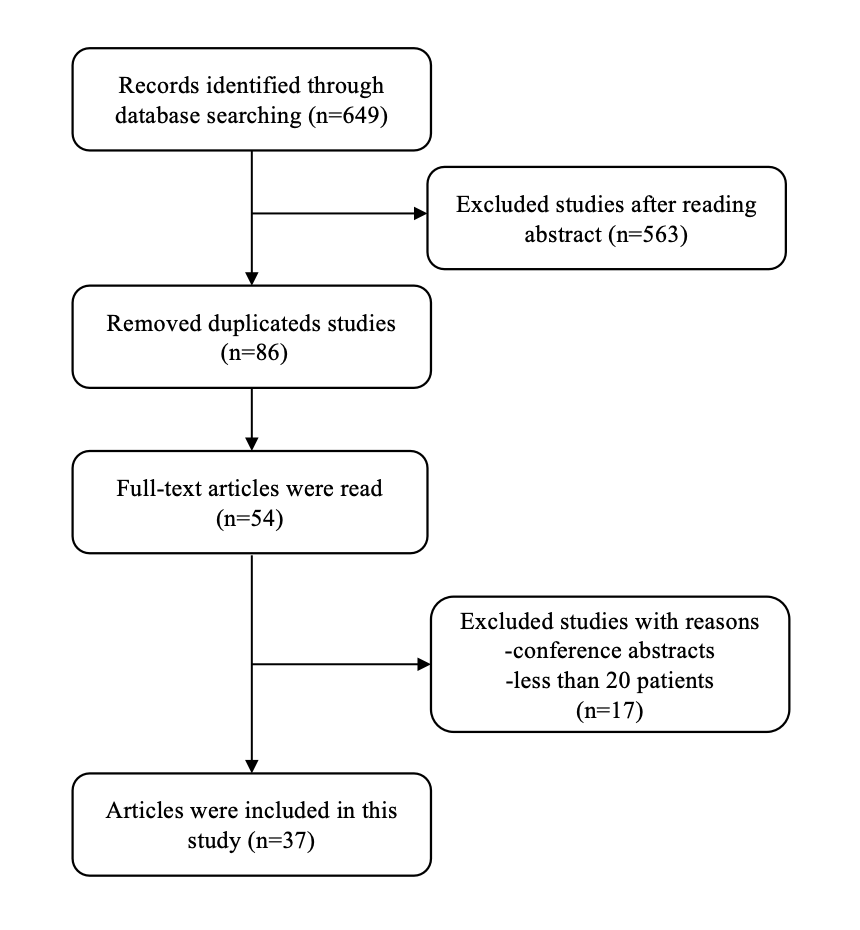

Supplement: Supplementary file 1 [file Table_1.DOCX]
